# The Over-Expression of an Arabidopsis B3 Transcription Factor, ABS2/NGAL1, Leads to the Loss of Flower Petals

**DOI:** 10.1371/journal.pone.0049861

**Published:** 2012-11-21

**Authors:** Jingxia Shao, Xiayan Liu, Rui Wang, Gaisheng Zhang, Fei Yu

**Affiliations:** 1 College of Life Sciences, Northwest A&F University, Yangling, Shaanxi, People’s Republic of China; 2 State Key Laboratory of Crop Stress Biology in Arid Areas (Northwest A&F University), Yangling, Shaanxi, People’s Republic of China; 3 College of Agronomy, Northwest A&F University, Yangling, Shaanxi, People’s Republic of China; Ohio State University, United States of America

## Abstract

Transcriptional regulations are involved in many aspects of plant development and are mainly achieved through the actions of transcription factors (TF). To investigate the mechanisms of plant development, we carried out genetic screens for mutants with abnormal shoot development. Taking an activation tagging approach, we isolated a gain-of-function mutant *abs2-1D* (*abnormal shoot 2-1D*). *abs2-1D* showed pleiotropic growth defects at both the vegetative and reproductive developmental stages. We cloned *ABS2* and it encodes a RAV sub-family of plant B3 type of transcriptional factors. Phylogenetic analysis showed that *ABS2* was closely related to *NGATHA* (*NGA*) genes that are involved in flower development and was previously named *NGATHA-Like 1* (*NGAL1*). *NGAL1* was expressed mainly in the root and the filament of the stamen in flower tissues and sub-cellular localization assay revealed that NGAL1 accumulated in the nucleus. Interestingly, over-expression of *NGAL1* driven by the constitutive 35S promoter led to transgenic plants with conspicuous flower defects, particularly a loss-of-petal phenotype. A loss-of-function *ngal1-1* mutant did not show obvious phenotype, suggesting the existence of redundant activities and also the utility of gain-of-function genetic screens. Our results show that the over-expression of NGAL1 is capable of altering flower petal development, as well as shoot development.

## Introduction

In eukaryotic organisms, gene expression regulations can occur at multiple levels to ensure the proper elaboration of the information stored in the genetic materials. Among the numerous factors that are involved in these intricate regulatory networks, transcription factors (TFs) play pivotal roles at the transcription level and they are intimately involved in many aspects of development [1]. Considering the central roles they play, it is not surprising to see the presence of large numbers of TFs in eukaryotic genomes. The model plant *Arabidopsis thaliana* genome contains more than 1500 transcription factors, accounting for ∼6% of its estimated ∼27,000 genes genome [2]. Typically, TFs contain distinct types of DNA-binding domains and transcriptional regulation regions and are capable of activating or repressing the expressions of a large number of target genes [3]–[6].

One family of transcription factors that has been under extensive investigation in plants is the plant-specific B3 superfamily TFs, which contain a characteristic ∼110 amino acids B3 domain responsible for DNA binding [7]. The B3 domain was originally named because it is the third basic domain in the maize protein VIVIPAROUS1 (VP1) [8]. In Arabidopsis and rice, there are at least 118 and 91 B3 family genes, respectively [7]. Arabidopsis B3 family of TFs can be further grouped into four subfamilies: ARF (AUXIN RESPONSE FACTOR), LAV (LEAFY COTYLEDON2 -ABSCISIC ACID INSENSITIVE3–VAL), RAV (RELATED TO ABI3 and VP1) and REM (REPRODUCTIVE MERISTEM) [7].

In Arabidopsis, the RAV subfamily consists of at least 13 members, including RAV1, RAV2/TEMPRANILLO2 (TEM2), TEM1, NGATHA1-4 (NGA1-4) and NGATHA-like 1–3 (NGAL1-3), and members of this subfamily of TFs have been implicated in many developmental and physiological processes in plants [9]. RAV1 and RAV2 were initially identified based on the B3 domain that they share with maize VP1 [10]. However, RAV1 and RAV2, as well as four other RAV subfamily members, contain a second DNA binding domain, the AP2 domain, which is the hallmark domain in AP2 family of TFs, in addition to the B3 domain, and both DNA binding domains are capable of binding DNA [10]. *RAV1* expression is down-regulated by the application of the phytohormone brassinosteroid and it may be a factor that negatively regulates leaf initiation, lateral root development and flowering transition [11]. RAV2/TEM2, as well as TEM1, may be regulators of flowering time and TEM1 can directly bind to *Flowering Locus T* (*FT*) promoter and negatively represses *FT* expression and flowering [12]. *RAV1* and *RAV2* expressions are also up-regulated by mechanical stimuli such as touch, wind, spray and transfer [13]. The NGATHAs and NGATHA-likes (NGA1-NGA4; NGAL1-NGAL3) are members of RAV subfamily that only contain the B3 DNA binding domain [9], [14]. The *nga1* mutant was isolated in genetic modifier screens in *pickle-15 kanadi1-2* (*pkl-15 kan1-2*) or *kan1-2 kan2-1/+* backgrounds, while *NGA3* was identified through the gain-of-function *tower-of-pisa1* (*top1*) mutant [9], [14]–[17]. Although single *nga1* mutants only exhibit subtle developmental phenotypes, they promote valve-like outgrowth, instead of the style-like outgrowth in gynoecium tissues in *pkl–15 kan1–2* or *kan1–2 kan2-1/+* backgrounds, suggesting that they are involved in the regulation of carpel polarity [9]. Quadruple *nga1 nga2 nga3 nga4* mutant exhibits a conversion of style to valve-like structures and this coincides with a reduced expression of the style-specific gene *SHATTERPROOF1* [9], [14]. Overall, one common theme seems to be that most, if not all, RAV subfamily members are involved in aspects of flower development.

Although much is now known about the functions of plant transcription factors and how they regulate plant growth and development, functions of many transcription factors remain poorly characterized. Novel investigation approaches, such as gain-of-function mutant screens, are offering new insights into TF functions [18]. We have identified numerous dominant gain-of-function mutants with altered shoot development and one of the semi-dominant mutants that we named *abs2-1D* (*abnormal shoot 2-1D*) was characterized here. *abs2-1D* displays a spectrum of phenotypes including a small plant stature, a faster leaf initiation rate, smaller leaves with abnormal shape and early flowering. We determined that the phenotypes of *abs2-1D* were the result of an elevated expression of *ABS2*, also called *NGAL1*, which is a RAV subfamily member of B3 transcription factor family. *NGAL1* was highly expressed in roots, flowers and siliques and its protein product is located in the cell nucleus. Interestingly, we found that the Cauliflower Mosaic Virus (CaMV) 35S promoter-driven *NGAL1* over-expression led to additional flower defects including a loss-of-petal phenotype. Our results suggest that NGAL1, when over-expressed, is capable of altering many facades of plant development.

## Results

### The Identification of *abs2-1D* Mutant

**Figure 1:**
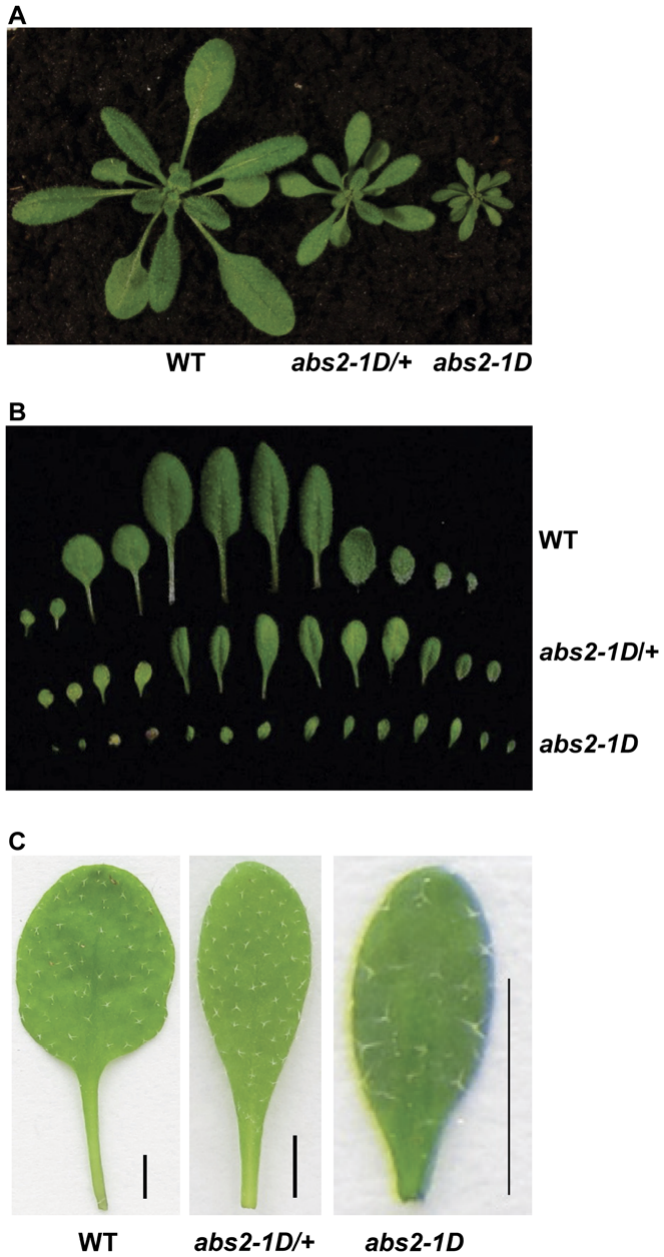
Phenotypes of *abs2-1D*. A. Three-week-old wild type, *abs2-1D*/+ heterozygous and *abs2-1D* homozygous plants. Plants were grown at 22°C under continuous illumination of ∼100 µmol·m^−2^·s^−1^. B. Cotyledons and rosette leaves of three-week-old wild-type, *abs2-1D*/+ heterozygous and *abs2-1D* homozygous plants. From left to right are two cotyledons and rosette leaves that were arranged in the order of their initiations. C. Comparison of the fifth rosette leaves of three-week-old wild type, *abs2-1D*/+ heterozygous and *abs2-1D* homozygous plants. Leaves were flattened between glass slides before photographing (Bars, 2 mm).

In our previous work looking for genetic suppressors of the Arabidopsis *yellow variegated* (*var2*) mutant, we carried out *var2* activation tagging mutagenesis [19]. Activation tagging is a modified T-DNA insertional mutagenesis: on one hand it can generate loss-of-function insertional mutants like the traditional T-DNA mutagenesis; on the other hand this procedure is also capable of producing gain-of-function mutants with the inclusion of four copies of the CaMV 35S enhancers near the right border of the T-DNA [18]. The main mechanism underlying activation tagging is the activation of transcriptions of genes adjacent to the T-DNA insertion site. In our large-scale screens for *var2* suppressors, we also identified a series of dominant mutants with altered shoot development that we named *abs* (*abnormal shoot*) mutants [20]. One semi-dominant mutant, *abnormal shoot2-1D* (*abs2-1D*; D for dominant), was characterized (Figure 1A). Both the heterozygous and the homozygous *abs2-1D* mutants were smaller in sizes than wild type (Figure 1A). One pronounced feature of the mutants was the abnormal leaf shape (Figure 1B–C). In wild type plants, there were clear distinctions between the leaf petiole and the leaf blade, while in *abs2-1D* the distinction between these two basic structures of a leaf was obscured and a gradual transition from petiole to blade was observed, and the leaves were also smaller (Figure 1B–C). In addition, *abs2-1D* mutant plants had a consistently higher leaf initiation rate at the vegetative stage (Figure 1A–B; Figure S1A). At the flowering stage, the heterozygous mutants developed shorter inflorescence than wild type and had reduced fertility, producing siliques that were much shorter than those of wild type (Figure S2A–B). The homozygous mutants were sterile under our growth conditions (Figure 1A).

**Figure 2:**
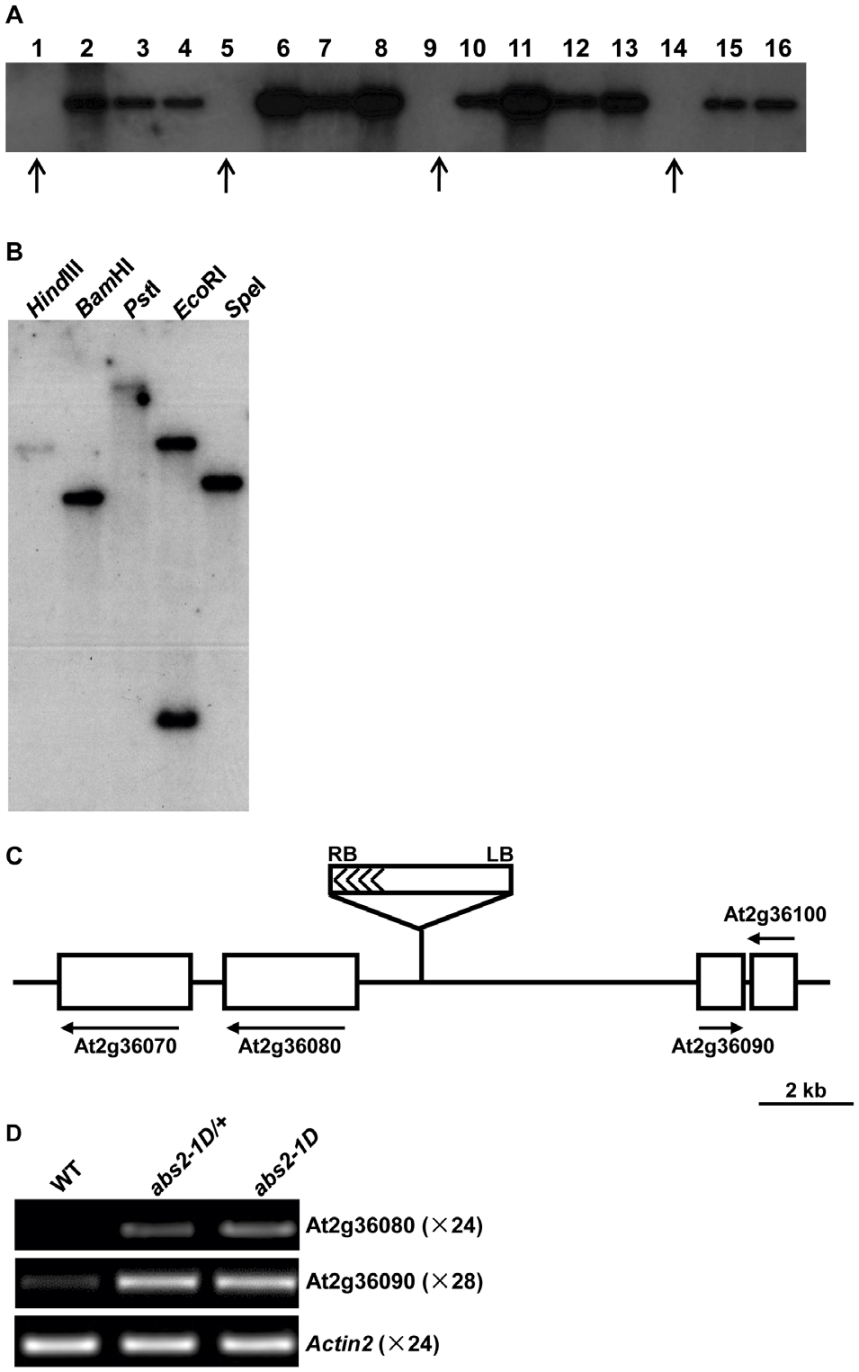
The cloning of *ABS2*. A. *abs2-1D* was genetically linked with T-DNA. Total leaf DNAs were extracted from 16 progenies of an *abs2-1D*/+ heterozygous plant. The DNAs were digested with *Hind*III and restriction fragments were separated with electrophoresis followed by transfer to a nylon membrane. The blot was probed with ^32^P labeled *BAR* gene sequences. Plants that did not show *abs2-1D* phenotypes were indicated by arrows. B. Confirmation of a single T-DNA insertion in *abs2-1D*. Genomic DNAs from *abs2-1D* plants were digested with indicated restriction enzymes. After electrophoresis and transfer to a nylon membrane, the blot was hybridized with ^32^P labeled *BAR* gene sequences. There is one *Eco*RI site in the probe sequence so two hybridizing bands were observed. C. Cloning of *abs2-1D*. In the *abs2-1D* mutant, activation tagging T-DNA was inserted between At2g36080 and At2g36090. Solid lines represent intergenic regions, while white boxes represent genes in the vicinity of the T-DNA insertion. The right border of the T-DNA was facing At2g36080. D. Semi-quantitative RT-PCR analysis of the expression levels of At2g36080 and At2g36090 in wild-type, *abs2-1D/+* heterozygous and *abs2-1D* homozygous mutants. *Actin2* expression was shown as a control. Total cellular RNAs were extracted from the aerial parts of two-week-old seedlings. 1 µg DNase I treated RNA from each sample was used for cDNA synthesis. RT-PCRs were performed with indicated numbers of cycles.

To investigate whether the phenotype of *abs2-1D* was linked with T-DNA insertion event(s), we carried out a co-segregation test. In a segregating population of *abs2-1D*, we performed southern blot analysis to see if T-DNA was associated with the *abs2-1D* phenotypes. Figure 2A shows that for all the plants that had the *abs2-1D* phenotypes, they also contained a T-DNA insertion, indicating that a T-DNA insertion is linked with *abs2-1D* phenotypes. Southern blot analysis with various restriction enzymes also indicated that the T-DNA insertion in *abs2-1D* was likely a single copy event (Figure 2B).

### The Cloning of *ABS2*

Since the *abs2-1D* phenotype was genetically linked with a single T-DNA insertion, we carried out plasmid rescue to identify the T-DNA insertion site in *abs2-1D*. We chose *Bam*HI as the enzyme because of its relatively smaller southern signal size (Figure 2B). Sequencing of the rescued plasmid showed that plant genomic DNA sequence outside the left border of the T-DNA was recovered and sequence analysis showed that the T-DNA was inserted between genes At2g36080 and At2g36090 on chromosome 2, with the T-DNA right border facing At2g36080. The physical distance between the insertion site and the start codon of At2g36080 was 1367 base pairs (Figure 2C). Given the semi-dominant nature of *abs2-1D* and the feature of activation tagging, it is likely that the genes flanking the T-DNA, particularly the gene outside of the right border, could be the target gene whose expression is elevated in the mutant. We tested the expressions of At2g36080 and At2g36090 in *abs2-1D* and semi-quantitative RT-PCR showed increased levels of At2g36080 and At2g36090 transcripts in both the *abs2-1D* heterozygous and homozygous mutant plants (Figure 2D).

### At2g36080 is *ABS2*

**Figure 3:**
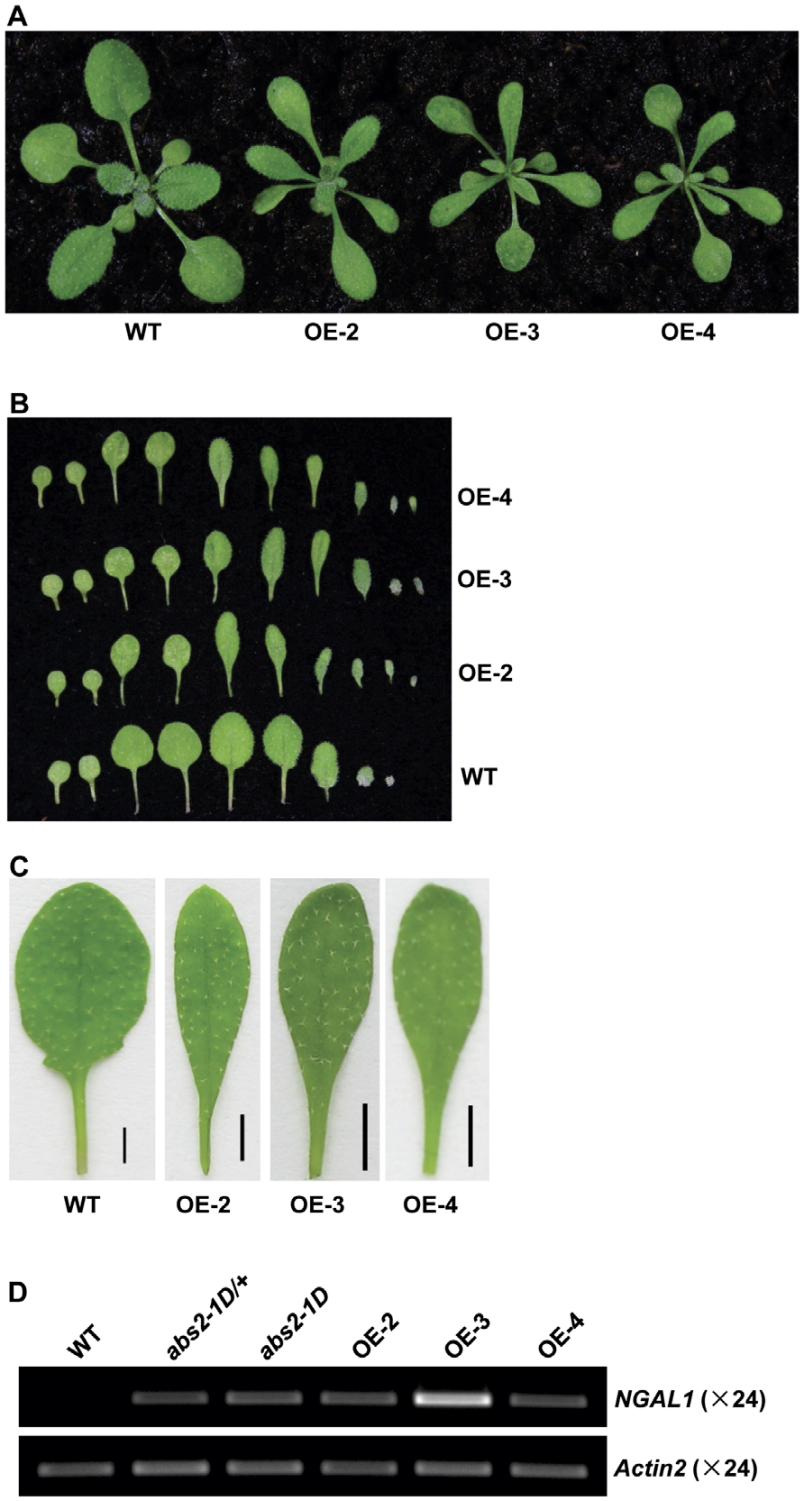
Phenotypes of *NGAL1* over-expression lines. A. Phenotypes of two-week-old wild type and three independent *NGAL1* over-expression (OE) lines, OE-2, OE-3 and OE-4. B. Cotyledons and rosette leaves detached from two-week-old wild-type, OE-2, OE-3 and OE-4 plants. From left to right were two cotyledons and rosette leaves that were arranged in the order of their initiations. C. Comparison of the fifth rosette leaf of two-week-old wild type, OE-2, OE-3 and OE-4 plants (Bars, 2 mm). D. Semi-quantitative RT-PCR analysis of the expression levels of *NGAL1* in wild-type, *abs2-1D*/+ heterozygous, *abs2-1D* homozygous, OE-2, OE-3 and OE-4 plants. RT-PCRs were carried out as in Figure 2D.

To confirm whether the over-expression of At2g36080 or At2g36090, was the cause behind the *abs2-1D* phenotypes, we carried out independent over-expression experiments. Full-length cDNA of At2g36080 was cloned into a binary vector and placed under the control of the CaMV 35S promoter. This vector was transformed into wild type Arabidopsis and transgenic At2g36080 over-expression (OE) plants were selected at T1 generation and allowed to self. At T2 generation, multiple At2g36080 OE transgenic lines showed leaf phenotypes similar to those of *abs2-1D*, in terms of the smaller plant size, the gradual leaf petiole to leaf blade transition and the faster initiation of leaves (Figure 3A–C; Figure S1B). In these lines, expressions of At2g36080 were increased to levels similar to or higher than those in *abs2-1D* (Figure 3D). These results indicate that the over-expression of At2g36080 is likely the cause for *abs2-1D* phenotypes and At2g36080 is *ABS2*.

### Phylogenetic Analysis

The ORF Finder analysis showed that the coding region of *ABS2*/At2g36080 was 735 bp in length, encoding a protein product of 244 amino acids. Sequence analysis revealed that ABS2 is a member of the RAV subfamily of the plant B3 transcription factors and is closely related to the NGATHA (NGA) proteins [9]. In a previous study, *ABS2*/At2g36080 was named *NGATHA-Like1* (*NGAL1*) and *ABS2*/At2g36080 will be referred to as *NGAL1* hereafter [9]. To further determine the evolutionary distance among the RAV proteins, phylogenetic analysis was carried out and Figure S3A shows that NGAL1 and NGA proteins were in the same clade, among the seven genes in the RAV sub-family that only contain the B3 domain. To support the phylogeny reconstruction, we compared intron and exon structures of all the *RAV* subfamily genes from Arabidopsis (Figure S3B). *RAV* genes have simple gene structures with most members only include one exon. Genes grouped in the clade including *NGAL1* have relatively complex gene structures of two or three exons (Figure S3B).

### Expression Profiles of *NGAL1*

**Figure 4:**
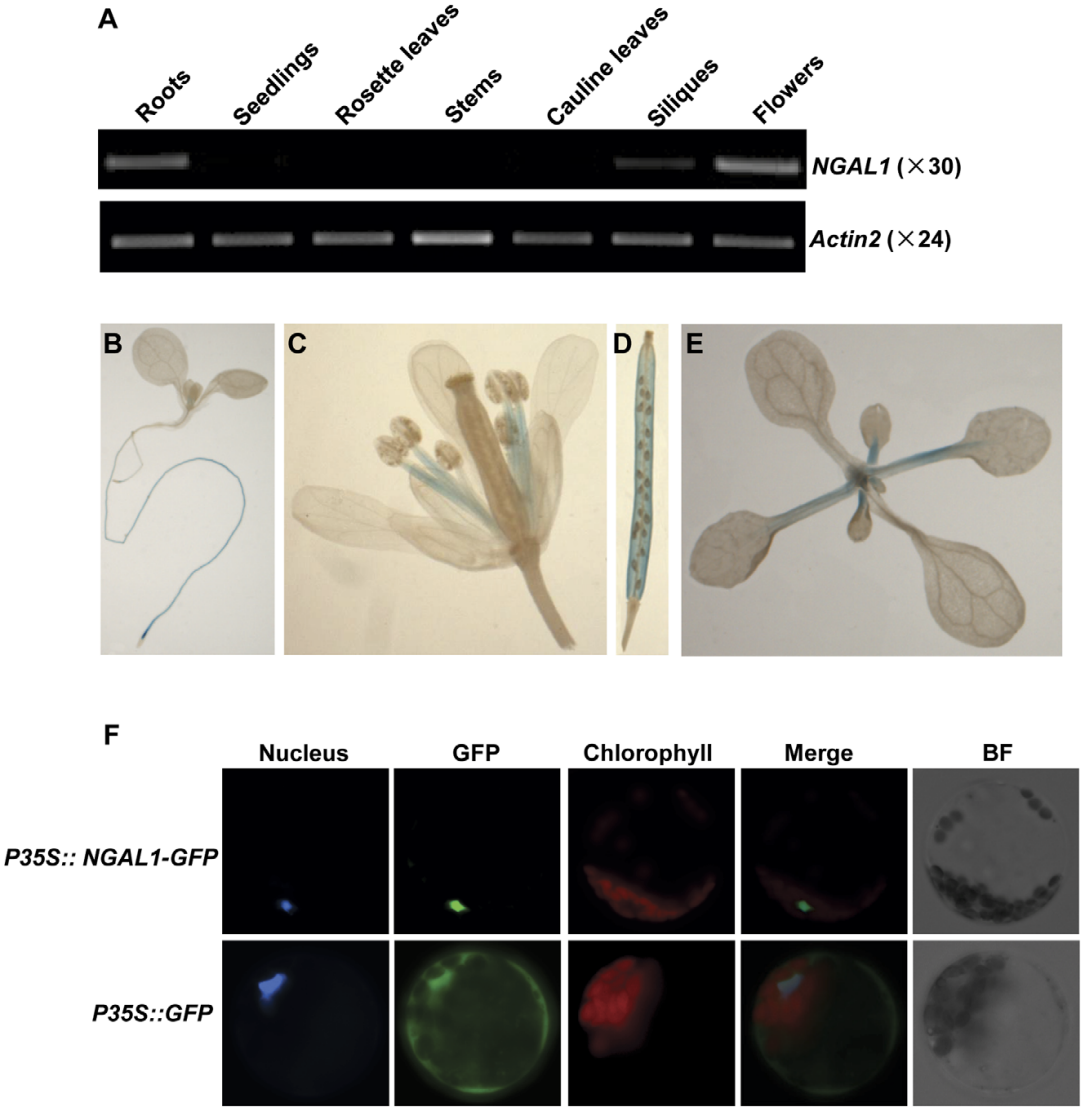
*NGAL1* tissue expression profile and NGAL1 protein localization. A. Expressions of *NGAL1* in different tissues of wild type plants were determined by semi-quantitative RT-PCR. Total RNAs were extracted from roots, two-week-old seedlings, rosette leaves, stems, cauline leaves, siliques and flower tissues and semi-quantitative RT-PCRs were carried out as in Figure 2D. *Actin2* expression was shown as a control. B–E. Tissue expression pattern of *NGAL1* examined by histo-chemical GUS staining. Illustrated are one-week-old seedling (B), flower (C), silique (D) and two-week-old seedling (E) from transgenic plants expressing *P_NGAL1_::GUS* fusion construct. F. Nuclear localization of *NGAL1-GFP* fusion protein in Arabidopsis leaf protoplasts. Nuclei of protoplasts were stained by Hoechst 33342. GFP fluorescence and bright field (BF) images of Arabidopsis protoplasts were compared to show the sub-cellular localization of GFP (cytosol and nucleus) and NGAL1-GFP (nucleus).

To examine the tissue expression pattern of *NGAL1*, we monitored its expressions in different Arabidopsis tissues including roots, two-week-old seedlings, rosette leaves, stems, cauline leaves, siliques, and flowers with semi-quantitative RT-PCR. Figure 4A shows that *NGAL1* was most highly expressed in the flower and root tissues. Although at lower levels, *NGAL1* transcripts were also present in siliques and two-week old seedlings (Figure 4A). Only very low levels of *NGAL1* expression were detected from tissues of rosette leaves, stems and cauline leaves (Figure 4A). Our results indicate that *NGAL1* expression shows some level of tissue specificity.

To further investigate the *NGAL1* expression profile, we constructed an *NGAL1* promoter-GUS fusion vector using a ∼1.2 kb promoter region upstream of its start codon. Wild type plants were transformed with this *P_NGAL1_-GUS* construct and GUS activities were assayed at T2 generation in six independent transgenic lines and they showed similar GUS activities (Figure 4B–E). Consistent with our RT-PCR results, we found major GUS activities in roots and flowers (Figure 4B–C). In the flowers, *NGAL1* expression was found primarily in the filament tissues of the stamen (Figure 4C). In the siliques, *NGAL1* was expressed in tissues including pericarp, but not in developing seeds (Figure 4D). In the vegetative tissues, *NGAL1* expression was found at leaf petioles (Figure 4E). Promoter-GUS assay further demonstrate the tissue specific expression profile of *NGAL1*.

### NGAL1 is Targeted to the Nucleus

To study the sub-cellular localization of NGAL1, the full-length *NGAL1* coding sequence was fused in-frame at its 3′ terminus with the GFP coding sequence to create a translational NGAL1-GFP fusion protein and the expression of this fusion protein was driven by the CaMV 35S promoter. The control vector contained GFP alone, which was also under the control of the 35S promoter. The two constructs were transformed and transiently expressed in Arabidopsis leaf protoplasts respectively. Fluorescent dye Hoechst 33342 was used to specifically indicate the nucleus [20], [21]. For the control vector, GFP green fluorescence signals were observed in both the cytosol and the nucleus (Figure 4F). In contrast, we observed NGAL1-GFP fluorescence signals that co-localized exclusively with the Hoechst 33342 fluorescence, suggesting that NGAL1 is a nuclear protein (Figure 4F).

### The Over-expression of *NGAL1* Leads to the Loss of Flower Petals

**Figure 5:**
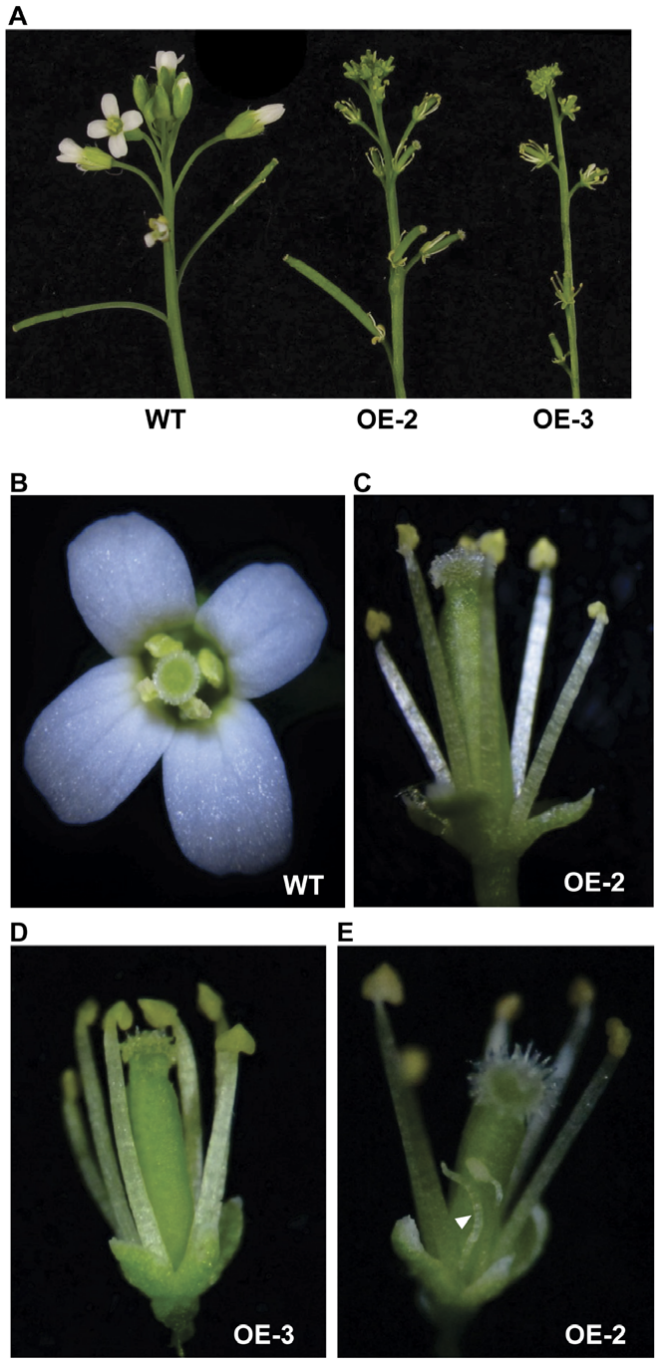
Flower phenotypes of *NGAL1* OE lines. A. Floral tissues of wild type and *NGAL1* OE plants. Note the conspicuous absence of the flower petals in *NGAL1* OE lines. B–E. Individual flower phenotypes of wild type and *NGAL1* OE plants. Individual flowers from wild type (B), two *NGAL1* OE lines, OE-2 (C and E) and OE-3 (D), were shown. Note the filamentous structure found in some flowers from OE lines (pointed by the white arrow head).

Upon closer examinations of the 35S promoter-driven *NGAL1* OE lines, we observed that multiple *NGAL1* OE lines showed pleiotropic development defects, in addition to the leaf phenotypes. Independent *NGAL1* OE lines, including OE-2 and OE-3, showed clear formations of inflorescence after 4 weeks of growth, when wild type plants were just starting to bolt (Table 1; Figure S4A). *NGAL1* OE lines also had more rosette leaves at bolting compared to wild type (Table 1). *NGAL1* OE lines produced flowers with reduced sizes and flower organs, including sepals, stamens and carpels were smaller in *NGAL1* OE lines compared with those of wild type (Figure 5A). Even more dramatic was the finding that strong *NGAL1* OE lines showed the absence of flower petals, the second whorl of the typical four whorls of Arabidopsis wild type flower organs (Figure 5A–D; Table 2). There were also intermediate petal phenotypes as we observed abnormal filamentous structures instead of petals in some flowers (Figure 5E; Table 2). We determined that *NGAL1* was indeed over-expressed in the petal-less flowers of independent OE lines, correlating the over-expression of *NGAL1* with the loss-of-petal phenotype (Figure S4B). Our results indicate that the over-expression of *NGAL1* is capable of altering flower development, particularly petal development in Arabidopsis.

**Table 1:** Comparison of the bolting times of wild type and *NGAL1* over-expression lines.

	Number of leavesat bolting	Bolting time (DAG)
wild type	16.88±0.72	27.62±0.60
OE-2	18.17±1.69, *p*<0.01	23.41±0.69, *p*<0.01
OE-3	18.93±0.70, *p*<0.01	23.84±1.51, *p*<0.01
OE-4	19.23±1.44, *p*<0.01	25.09±1.06, *p*<0.01

The average numbers of leaves of wild type and OE lines at bolting were calculated from randomly selected plants of each genotype (n≥28). Bolting times were calculated as days after germination (DAG). Data were presented in the form of mean±standard deviation (s.d.). Comparisons were made between wild type and each of the OE lines. Statistical significance was evaluated by *p* values generated by Student’s *t-*test.

**Table 2:** Quantification of the loss-of-petal phenotype in *NGAL1* over-expression lines.

OE Lines	Total^1^	Flowers w/Four Normal Petals	Abnormal Flowers^2^						Abnormality
			No Petal	One Abnormal Petal	Two Abnormal Petals	Three Abnormal Petals	Four Abnormal Petals		
OE-1	160	13 (8.1%)	40 (25%)	43 (26.9%)	39 (24.4%)	16 (10%)	9 (5.6%)	91.9%	
OE-2	206	7 (3.4%)	56 (27.2%)	61 (29.6%)	51 (24.8%)	13 (6.3%)	18 (8.7%)	96.6%	
OE-3	136	3 (2.2%)	36 (26.5%)	38 (27.9%)	30 (22.1%)	21 (15.4%)	8 (5.9%)	97.8%	
OE-4	182	8 (4.4%)	32 (17.6%)	58 (31.9%)	62 (34.1%)	18 (9.9%)	4 (2.2%)	95.6%	
OE-9	122	13 (10.7%)	22 (18.0%)	42 (34.4%)	28 (23.0%)	9 (7.4%)	8 (6.6%)	89.3%	

Plants were randomly selected from each OE line (N≥28) to score the petal loss phenotype. Flowers produced by each plant were randomly picked and examined with a stereoscope.

1 Total numbers of flowers examined for each OE line.

2 Abnormal flowers are defined as flowers have at least one abnormal filament-like petal or petal less.

### Identification of a *NGAL1* Loss-of-function Mutant Allele

**Figure 6:**
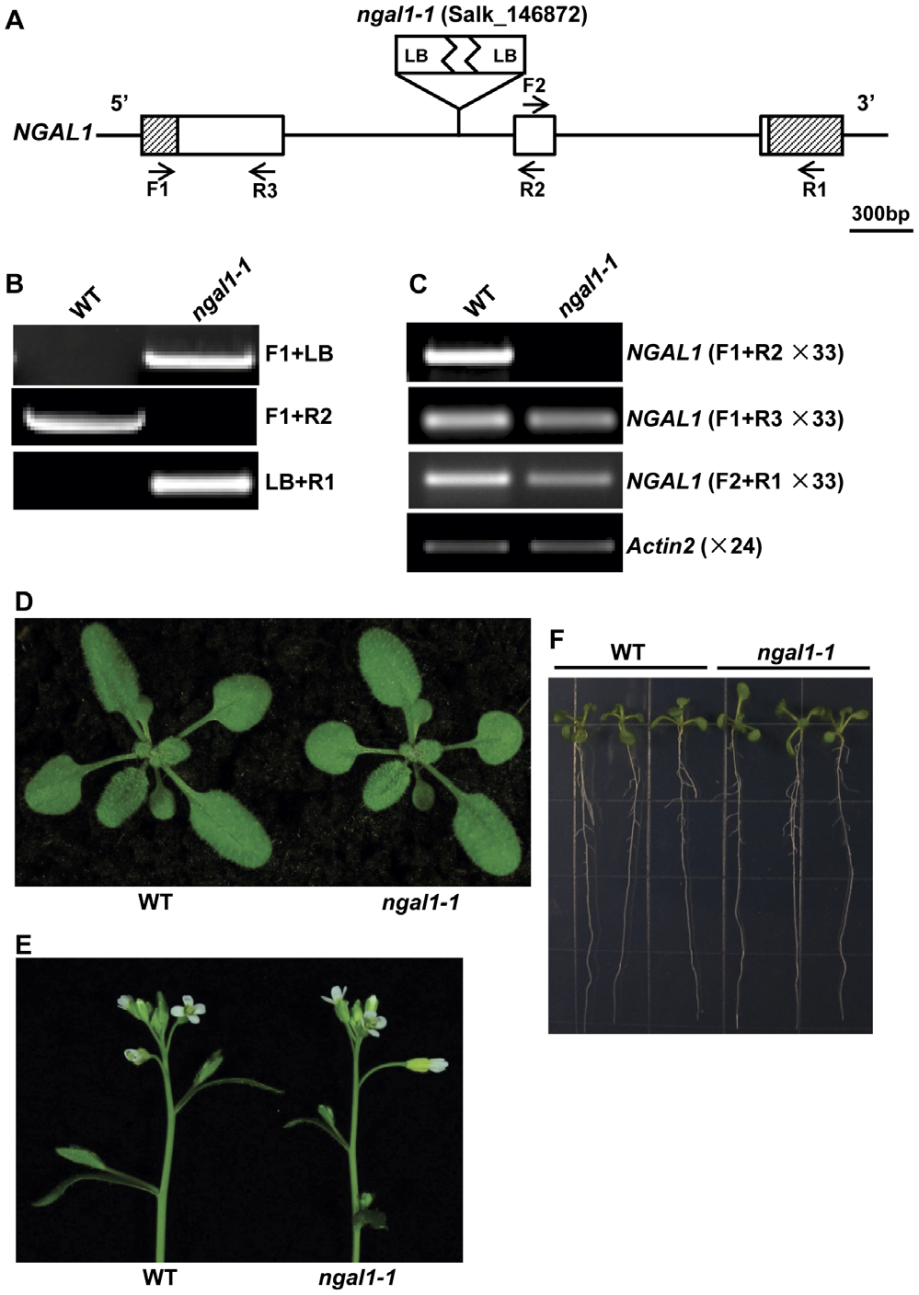
Identification of a loss-of-function mutant allele of *NGAL1*. A. T-DNA insertion site in Salk_146872 (*ngal1-1*). Lines represented introns and intergenic regions and boxes represented exons. 5′ and 3′ UTRs were indicated by shaded boxes. Approximate positions of the PCR primers used in B and C were marked with arrows. B. PCR-identification of *ngal1-1* homozygous mutant. The T-DNA insertion was flanked by two LB sequences. C. Expressions of *NGAL1* in wild type and *ngal1-1* mutant. Total RNAs were extracted from flower tissues and RT-PCRs were carried out with indicated primers and cycle numbers. D. Phenotypes of two-week-old wild type and *ngal1-1* homozygous seedlings. E. Floral tissues of five-week-old wild type and *ngal1-1* homozygous plants. F. Comparison of root phenotypes of one-week-old wild type and *ngal1-1* homozygous plants.

The phenotypes we observed with *abs2-1D* and *NGAL1* OE lines were the consequences of gain-of-function genetic manipulations. To investigate potential loss-of-function phenotypes of *NGAL1*, we searched for its T-DNA insertional mutants in The Arabidopsis Information Resource (TAIR) and identified a putative insertion line SALK_146872 (Figure 6A). PCR analysis confirmed that the T-DNA was inserted in the first intron of *NGAL1* (Figure 6B). We also found that the inserted T-DNA structure likely included two copies of T-DNAs because we identified both ends of the T-DNA insertion as the left border (Figure 6B). Homozygous line of SALK_146872 (named *ngal1-1*) was identified and RT-PCR results showed that full-length *NGAL1* cDNAs cannot be detected in the floral tissues, where *NGAL1* was normally expressed (Figure 6B–C). However, we did detect the presence of transcripts using primer sets that do not span the T-DNA, indicating that certain forms of abnormal transcripts of *NGAL1* may exist in *ngal1-1* (Figure 6C). Although *NGAL1* over-expression led to the loss of flower petals, loss-of-function *ngal1-1* plants showed no visible phenotypic changes at vegetative stages when compared with the wild type plants under our growth conditions (Figure 6D). We also did not observe abnormalities with *ngal1-1* roots and flowers (Figure 6E–F). Our results suggest that the absence of *NGAL1* does not dramatically alter plant development and its activity is likely buffered by redundant genes or activities.

## Discussion

### The Over-expression of *ABS2*/*NGAL1* is Capable of Altering Multiple Aspects of Plant Development

We are interested in exploring the genetic regulatory networks that govern the growth and development of higher plants. Taking a forward genetics approach, we screened for mutants with abnormal shoot development phenotypes in activation tagging mutant populations and investigated these *abnormal shoot* (*abs*) mutants further [20]. Here, we report the isolation and characterization of one semi-dominant *abs* mutant, *abs2-1D*, which has multiple developmental defects at both the vegetative and reproductive stages. The cloning of *ABS2* revealed that it encodes a member of the RAV subfamily of the plant B3 family of transcription factors. Four major classes of transcription factors containing the B3 domain have been identified in plants, including the LAV, ARF, RAV, and REM subfamilies [7]. The 13 RAV subfamily members can be further placed into two groups based on the features of their DNA binding domains. ABS2, one of the seven RAV subfamily members that only have the B3 DNA binding domain, is closely related to *NGA* genes and is also named *NGAL1* [9]. The other six members contain both the B3 domain and an additional AP2 DNA binding domain [22]. Transcription factors containing the B3 domain have been shown to be responsive to biotic and abiotic stresses, as well as to phytohormones such as abscisic acid, auxin and brassinosteroids [11], [23], [24]. Members of plant RAV subfamily are involved in multiple development programs including flower development [9], [14]–[17]. Two independent efforts have established that the *NGA1–4* genes regulate style development [9], [14].

In both the *abs2-1D* mutant and the independent 35S-driven *NGAL1* OE lines, shoot development programs were clearly altered. We also found that multiple 35S promoter-driven *NGAL1* OE lines showed the loss-of-petal phenotype, suggesting that *NGAL1* over-production can alter flower development. We did not observe the loss-of-petal phenotype in the *abs2-1D* mutant and one possibility is that the over-expression mechanisms were slightly different. In *abs2-1D*, over-expression was through the activation of *NGAL1* expression by the presence of 35S enhancer sequences, whereas the intact 35S promoter was used in 35S promoter-driven over-expression. Phenotypes of our OE lines are also somewhat different from a previous OE of *NGAL1*, in which a different promoter was used [9]. However, we cannot rule out the possibility that the presence of 35S enhancers in *abs2-1D*, which can activate the expressions of many genes in their vicinity, might contribute to the difference.

Our RT-PCR results indicated that *NGAL1* is normally expressed in many plant tissues including roots, flowers, siliques and young seedlings. Further promoter-GUS assay revealed that *NGAL1* expressions also show clear tissue-specific patterns with the highest expressions in roots and the filament of the stamen. These data suggest *NGAL1* functions are necessary in different tissues and it may be involved in many developmental processes in plants. Because NGAL1 is predicted to be a transcription factor and we have shown that it is targeted to the nucleus, it is likely NGAL1 regulates plant development in the nucleus, possibly through modulating the expressions of its target genes, directly or indirectly.

### *NGAL1* Over-expression Leads to the Loss of Flower Petals

In Arabidopsis, there are four whorls of floral organs: sepals, petals, stamens and carpels. The identities of floral organs are controlled by at least five classes of homeotic genes (*ABCDE* genes), and mutations in these floral regulators lead to homeotic conversions from one to another and work in this area has led to the establishment of the ABC model as the basis for the formation of floral organs [25].

During flower development, petal identity is determined by the combination of class A (*AP1*, *AP2*), class B (*AP3*, *PI*) and class E (*SEP*) genes [25], [26]. In weak alleles of homeotic mutants such as *ap2* and *ap3*, if petal primordia are able to initiate, they are converted into stamens and sepals, respectively as primordia develop further [27], [28]. Another set of genes have been implicated in a different aspect of petal development, more specifically, petal initiation [29]–[34]. The Arabidopsis *petal loss* (*ptl*) mutant was isolated because of the loss-of-petal phenotype [29]. For the occasional petals that do develop, the orientations of petals are also disturbed in the *ptl* mutant [29]. *PTL* encodes a tri-helix transcription factor [30]. The *rabbit ears* mutant displays similar petal loss phenotypes to *ptl* and *RABBIT EARS* (*RBE*) encodes a zinc finger protein that is located in the nucleus and shares homology with SUPERMAN [31]. *RBE* may regulate second whorl initiation through the repression of *AGAMOUS* [32]. A third Arabidopsis gene, *ROXY1*, which encodes a glutaredoxin protein, is also necessary for petal initiation [33]. Interestingly, in all three cases, the petal phenotypes are position dependent, rather than identity dependent. Other factors, including UNUSUAL FLORAL ORGANS (UFO), have also been implicated in the petal development [34]. The exact mechanisms that these factors regulate petal initiation remain unclear.

Through the over-expression of *NGAL1*, we found that transgenic *NGAL1* OE plants display flower defects, particularly an intriguing loss-of-petal phenotype, suggesting that the over-production of *NGAL1* has the capacity to dramatically alter the normal development of flower petals. In contrast to the above-mentioned recessive mutations that lead to the loss-of-petal phenotype, our findings establish a case where a dominant gain-of-function mutation confers the loss-of-petal defect, similar to the over-expression of *AG* [35]. Based on the dominant nature, it can be argued that *NGAL1*, when over-expressed, may function as a negative regulator of petal initiation or petal development or both. Consistent with this notion of NGAL1 being a negative regulator, a recent report has shown that NGAL1 might function as a transcriptional repressor [36]. The impact of *NGAL1* over-expression is not limited to flower development as its over-production also causes changes of leaf shape and the rate of leaf initiation. Our findings suggest that *NGAL1*, when over-expressed, is capable of impacting many aspects of plant development. It is important to point out that the phenotypes we observed with *NGAL1* gain-of-function over-expression studies do not match completely with the tissue expression pattern of *NGAL1*. For example, the highest *NGAL1* expression in the wild type flower is in the filament of the stamen but the most conspicuous flower defect in *NGAL1* OE plants is the loss of petals. Although we cannot rule out that *NGAL1* transcripts are present at low levels in other parts of the flower or in other stages of flower development, our findings suggests that NGAL1 may regulate plant development outside of its normal expression domains and this capacity offers a new gene resource for engineering plants with desirable traits such as flowers without the petals.

To further our understanding of *NGAL1* functions, we also identified a loss-of-function *NGAL1* mutant allele. However, we did not observe major developmental defects in this mutant, despite the reduction of *NGAL1* expression. The lack of obvious phenotypes in the *NGAL1* loss-of-function mutant is not entirely unexpected given the presence of multiple homologous genes in the RAV subfamily that might provide redundant functions [14]. However, this superficial lack-of-phenotype does not necessarily mean there are no subtle changes in the mutant that may only be manifest under certain circumstances, such as in another mutant’s background. For example, single mutants of *NGA* genes only show subtle mutant phenotypes but can cause dramatic floral defects in certain mutant backgrounds [9].

## Materials and Methods

### Plant Materials and Growth Conditions

The wild-type *Arabidopsis thaliana* and the *abs2-1D* mutant used in this study were of the Columbia-0 (Col) ecotype. The T-DNA insertion mutant (Salk_146872C) seeds were obtained from TAIR. Seeds were sown on commercial peat moss mix (Pindstrup, Denmark) and stratified at 4°C for 2 days. Plants were grown at 22°C under continuous illumination of ∼100 µmol·m^−2^·s^−1^.

### Phylogenetic Analysis

To generate the phylogenetic tree of the RAV subfamily proteins, full-length amino acid sequences were retrieved from TAIR. Protein sequence alignment was carried out using the CLUSTALW software (http://www.ebi.ac.uk/Tools/clustalw2/). Phylogeny construction, bootstrap test of phylogeny by means of the neighbor-joining method and the Poisson correction model were performed using MEGA software version 4.0 [37]. Bootstrap analysis was performed using 1000 trials, and At2g30470 (VAL1) was used as an outgroup.

Genomic and coding sequences of RAV subfamily members were obtained from TAIR and used for the construction of gene structures.

### Vector Constructions and Plant Transformation

To construct a *NGAL1* over-expression vector, the full length At2g36080 cDNA was amplified with primers 36080F (5′-CATGGATCCTCTCTCATCACTATTTGCCATCTC-3′) and 36080R (5′-CATGGATCCCATCTATGACAACATAACAGGACC-3′) and ligated into pBluescript. After confirming the correct sequences, the cDNA was subcloned into a binary vector pBI111L and placed under the control of the CaMV 35S promoter. To generate *ABS2* promoter-GUS fusion construct, a genomic fragment of ∼1.2 kb upstream of the start codon of At2g36080 was amplified with primers 36080PF (5′-CATTCTAGAAGAGGATTGAAACACGACTGTAGT-3′) and 36080PR (5′-CATGGATCCTTGGCTAGGTTACATGTATCTGC-3′) and cloned into a binary vector pCB308 [38]. Transgenic plants were generated by *Agrobacterium tumefaciens*-mediated floral dip method [39]. T1 seeds were screened on half-strength Murashige and Skoog (MS) solid medium containing 50 mg·L^−1^ kanamycin (for OE lines) or on soil for Basta resistance (for promoter-GUS lines). Histochemical GUS staining was carried out as described in [20]. GUS activities were examined in multiple independent transgenic lines in T1 and T2 generations.

### Morphological Observations

Phenotypes of Arabidopsis flowers were observed and photographed using an Olympus SZ61 stereomicroscope equipped with a Canon G12 camera and greenhouse-grown Arabidopsis plants were photographed directly with a Canon G12 camera.

To quantify the leaf initiation rates of wild type, *abs2-1D* mutants and *NGAL1* OE lines, numbers of rosette leaves were counted from randomly selected plants (n≥28) of each genotype on a daily basis starting from one-week-old plants until bolting. Mean and standard deviation of leaf numbers were calculated.

To quantify the loss-of-petal phenotype of *NGAL1* OE lines, randomly picked flowers from each OE line were sorted into two categories first: normal flowers (defined as flowers with four normal shaped petals) and abnormal flowers (defined as flowers with filament-like abnormal petals or without petals). Percentage of each type of flowers and the total percentage of abnormality of each OE line were calculated.

### DNA and RNA Manipulations

Genomic DNAs were isolated and Southern-blot analyses were performed as described in Yu et al [19].

Total cellular RNAs were extracted with Trizol Reagents (Invitrogen, USA) according to the manufacturer’s instructions and stored at −80°C. First-strand cDNA was synthesized with 1 µg of total RNA using PrimeScript™ II 1st strand cDNA synthesis Kit (Takara, Japan). Primers 36080F1 (5′-TCATCACTATTTGCCATCTC-3′), 36080R1 (5′-CTATGACAACATAACAGGAC-3′), 36080F2 (5′-AACCAATCACGACCAGTTTC-3′), 36080R2 (5′-AAACTGGTCGTGATTGGTTG-3′) and 36080R3 (5′-TGAACATGGCGATAAGAGTC-3′) were used for detecting At2g36080 transcripts. *Actin2* gene expression was monitored using the primers ACT2F (5′-TCAAAGACCAGCTCTTCCATCGAGA-3′) and ACT2R (5′-ACACACAAGTGCATCATAGAAACGA-3′). At2g36090 transcripts were amplified with primers 36090F1 (5′-ACGACATCATAGAGTCTCAC-3′) and 36090R1 (5′-TCTACCTTGAGACTCACTTC-3′).

### Transient Expression of NGAL1-GFP in Arabidopsis Protoplasts

To construct the *NGAL1-GFP* fusion gene, the coding region of *NGAL1* was amplified by PCR, using primers ABS2-F1 (5′-CATGGGATCCTCATCACTATTTGCCATCTC-3′) and ABS2-GFPR (5′-CATGGGATCCACCACCACCACCACCACCGCTCGTCCGGTTCATATCTCC-3′). The resulting fragment was digested with *Bam*HI and ligated in frame with the GFP coding sequence in the pTF486 vector and sequenced [19]. Arabidopsis leaf protoplast isolation and Hoechst 33342 nucleus staining were performed as described in [20], [21]. Fluorescence and bright field images were captured and analyzed with fluorescence microscopy (DM5000B, Leica, Germany).

### Isolation of *ngal1-1*

To isolate *NGAL1* T-DNA mutant (*ngal1-1*), we obtained a putative T-DNA insertion mutant line (Salk_146872C) from TAIR. Genomic DNAs were prepared and gene-specific primers, 36080F1, 36080R2, and T-DNA specific primers LB (5′-GAACAACACTCAACCCTATCTC-3′) were used to identify heterozygous and homozygous plants. PCR products were gel purified and sequenced.

## Supporting Information

Figure S1 **Comparison of leaf initiation rates of wild type,** ***abs2-1D*** **mutants and** ***NGAL1*** **overexpression lines.**
(PDF)

Figure S2 **Phenotypes of** ***abs2-1D*** **mutants at flowering stages.**
(PDF)

Figure S3 **The Arabidopsis RAV sub-family of transcription factors.**
(PDF)

Figure S4 **Phenotypes of** ***NGAL1*** **OE lines at flowering stage.**
(PDF)
