# Supplementary material for: The Over-Expression of an Arabidopsis B3 Transcription Factor, ABS2/NGAL1, Leads to the Loss of Flower Petals
Source: PLoS One. 2012 Nov 21;7(11):e49861. doi: 10.1371/journal.pone.0049861 (PMC3503873; doi:10.1371/journal.pone.0049861)
Supplement: Figure S1 — Comparison of leaf initiation rates of wild type, abs2-1D mutants and NGAL1 overexpression lines. (PDF) [file pone.0049861.s001.pdf]

Figure S1

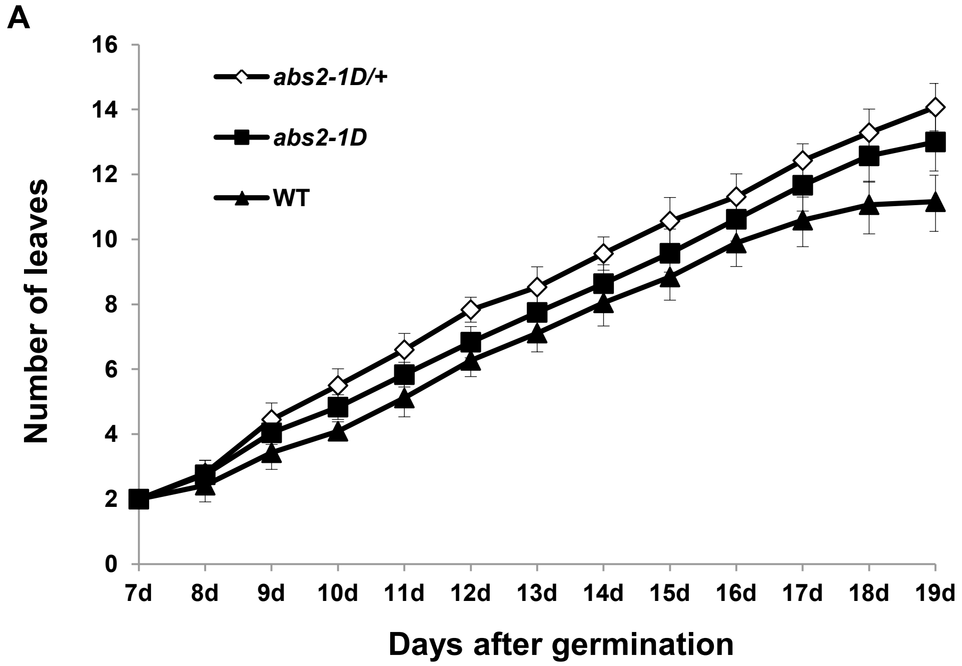

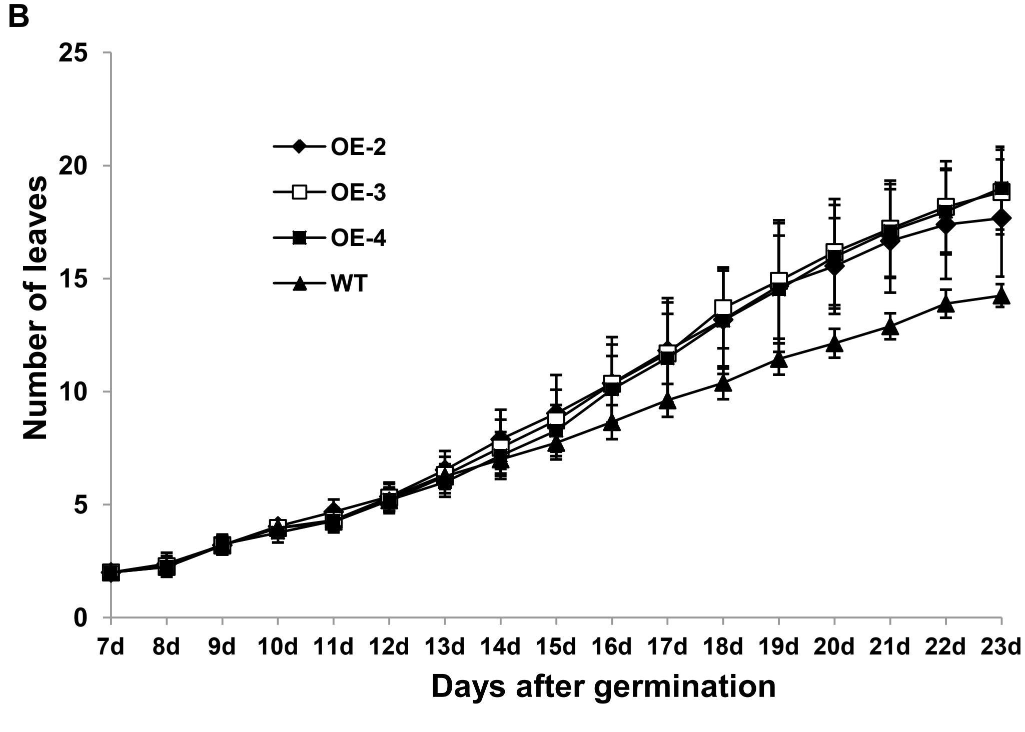

Figure S1. Comparison of leaf initiation rates of wild type, *abs2-1D* mutants and *NGAL1* over-expression lines.

Numbers of rosette leaves were counted from randomly selected plants (n ≥28) of each genotype on a daily basis starting from one-week-old plants till bolting. Average leaf numbers were calculated. Error bars represent ± standard deviation (s.d.). A. Leaf initiation rates for wild-type, *abs2-1D/+* heterozygotes and *abs2-1D* homozygotes. B. Leaf initiation rates for wild-type and three independent *NGAL1* over-expression lines (OE-2, OE-3 and OE-4).
