# Supplementary material for: The Over-Expression of an Arabidopsis B3 Transcription Factor, ABS2/NGAL1, Leads to the Loss of Flower Petals
Source: PLoS One. 2012 Nov 21;7(11):e49861. doi: 10.1371/journal.pone.0049861 (PMC3503873; doi:10.1371/journal.pone.0049861)
Supplement: Figure S2 — Phenotypes of abs2-1D mutants at flowering stages. (PDF) [file pone.0049861.s002.pdf]

Figure S2

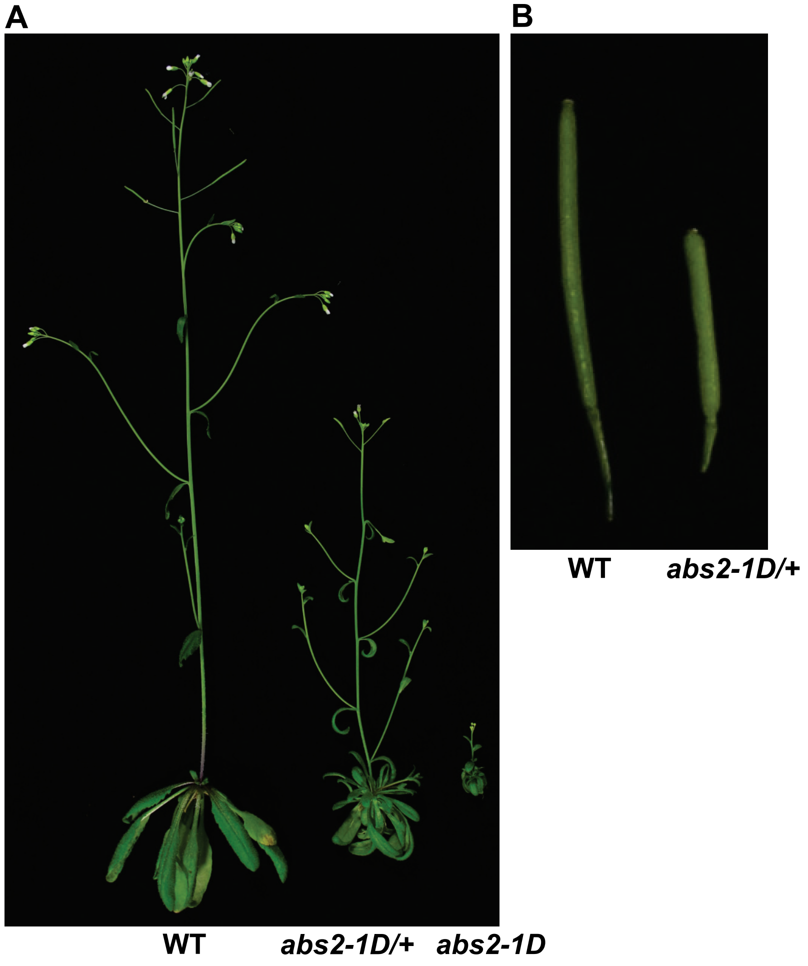

Figure S2. Phenotypes of *abs2-1D* mutants at flowering stages.

A. Phenotypes of five-week-old wild type, *abs2-1D/+* heterozygote and *abs2-1D* homozygote plants.

B. Representative siliques of wild type and *abs2-1D/+* heterozygote.
