# Supplementary material for: The Over-Expression of an Arabidopsis B3 Transcription Factor, ABS2/NGAL1, Leads to the Loss of Flower Petals
Source: PLoS One. 2012 Nov 21;7(11):e49861. doi: 10.1371/journal.pone.0049861 (PMC3503873; doi:10.1371/journal.pone.0049861)
Supplement: Figure S3 — The Arabidopsis RAV sub-family of transcription factors. (PDF) [file pone.0049861.s003.pdf]

Figure S3

A

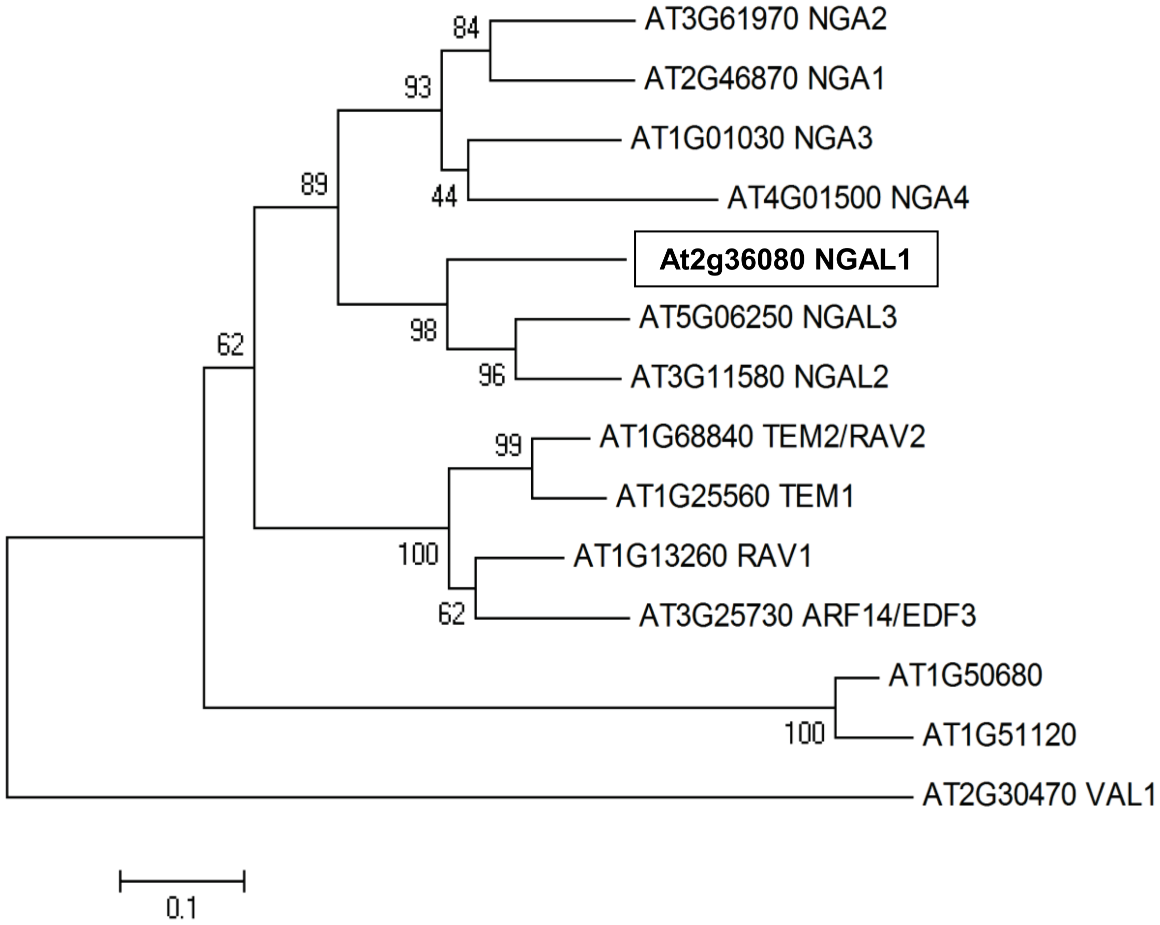
AT3G61970 NGA2
AT2G46870 NGA1
AT1G01030 NGA3
AT4G01500 NGA4
At2g36080 NGAL1
AT5G06250 NGAL3
AT3G11580 NGAL2
AT1G68840 TEM2/RAV2
AT1G25560 TEM1
AT1G13260 RAV1
AT3G25730 ARF14/EDF3
AT1G50680
AT1G51120
AT2G30470 VAL1
84
93
44
89
98
96
62
99
100
62
100
0.1

Figure S3

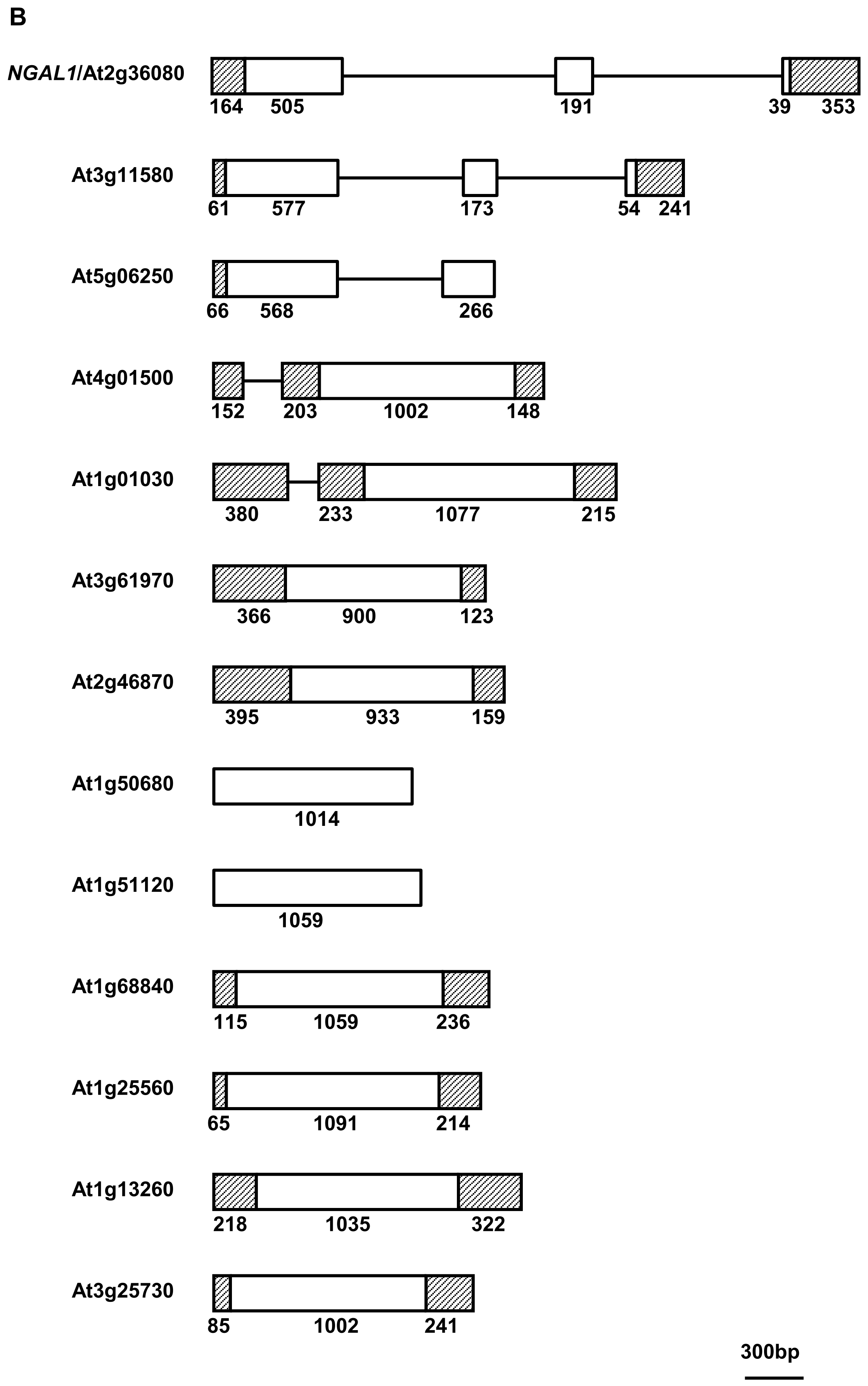
B
NGAL1/At2g36080
164
505
191
39
353
At3g11580
61
577
173
54
241
At5g06250
66
568
266
At4g01500
152
203
1002
148
At1g01030
380
233
1077
215
At3g61970
366
900
123
At2g46870
395
933
159
At1g50680
1014
At1g51120
1059
At1g68840
115
1059
236
At1g25560
65
1091
214
At1g13260
218
1035
322
At3g25730
85
1002
241
300bp

Figure S3. The *Arabidopsis* RAV sub-family of transcription factors.

A. Phylogenetic analysis of the RAV proteins in *Arabidopsis*. VAL1 (At2g30470) was used as an outgroup, and numbers represent bootstrap percentage from 1000 trials.

B. Gene structures of RAV sub-family of transcription factor in *Arabidopsis*. Lines represented introns and boxes represented exons. 5' and 3' UTRs were indicated as shaded boxes. All genes structures were presented in the 5' to 3' direction. The number of nucleotides in UTRs and exons were also indicated.
