# Supplementary material for: The Over-Expression of an Arabidopsis B3 Transcription Factor, ABS2/NGAL1, Leads to the Loss of Flower Petals
Source: PLoS One. 2012 Nov 21;7(11):e49861. doi: 10.1371/journal.pone.0049861 (PMC3503873; doi:10.1371/journal.pone.0049861)
Supplement: Figure S4 — Phenotypes of NGAL1 OE lines at flowering stage. (PDF) [file pone.0049861.s004.pdf]

Figure S4

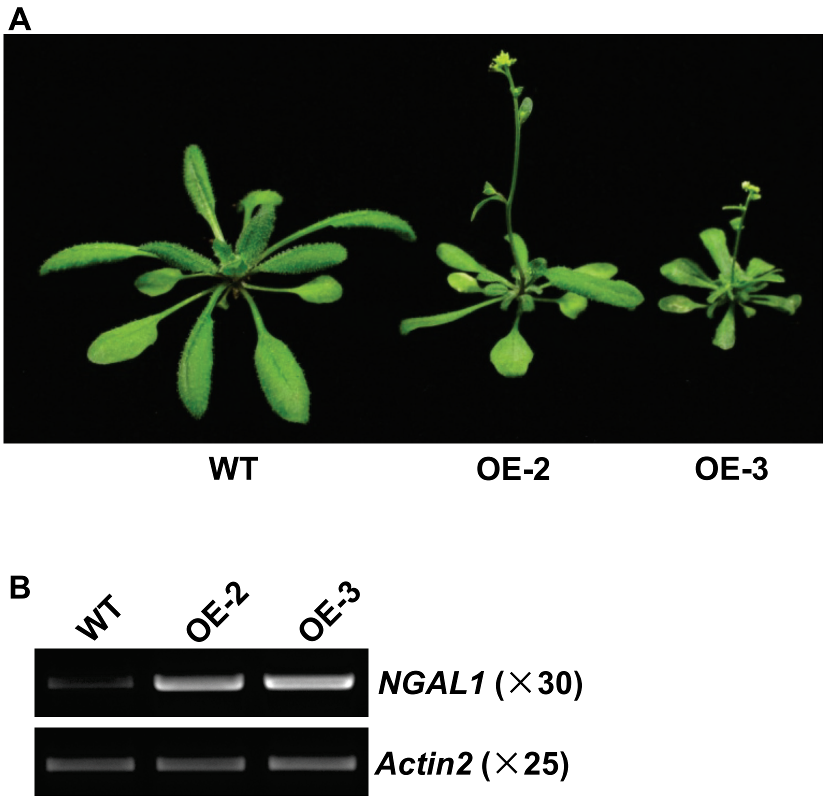

Figure S4. Phenotypes of *NGAL1* OE lines at flowering stage.

A. Illustrated are representative four-week-old wild type, *NGAL1* OE-2 and OE-3 plants.

B. Semi-quantitative analysis of *NGAL1* transcripts accumulation in wild type and *NGAL1* OE lines. Total cellular RNAs were extracted from wild type flowers and from petal-less flowers of OE lines. RT-PCRs were performed as in Figure 2D with indicated cycle numbers.
